# Complete Genomic Sequences of Two Agarolytic *Vibrio* Species Isolates from the Red Algae *Gracilaria*

**DOI:** 10.1128/mra.00934-22

**Published:** 2022-11-07

**Authors:** Mei Ito, Mikihisa Muta, Takashi Funatsu, Yuji Hatada, Ryo Iizuka

**Affiliations:** a Department of Life Science and Green Chemistry, Faculty of Engineering, Saitama Institute of Technology, Saitama, Japan; b Graduate School of Pharmaceutical Sciences, The University of Tokyo, Tokyo, Japan; c Department of Biological Sciences, Graduate School of Science, The University of Tokyo, Tokyo, Japan; Montana State University

## Abstract

We report the complete genomic sequences of two agarolytic *Vibrio* species strains, STUT-A11 and STUT-A16, isolated from the red algae *Gracilaria*. Genomic annotations revealed that both strains harbor four β-agarases, α-neoagarooligosaccharide hydrolase, and agarolytic β-galactosidase, which support efficient agarose catabolism.

## ANNOUNCEMENT

Agarose is a red algal carbohydrate potentially valuable for producing physiologically active agarooligosaccharides, biofuels, and other chemicals (1). Agarolytic *Vibrio* species isolates were enriched in the basal medium (artificial seawater [Gex, Osaka, Japan], 0.5% yeast extract, and 0.1% NH_4_Cl) containing a portion of the red algae *Gracilaria* from the Japanese sea coast at Yamaguchi Prefecture, Japan (latitude, 34.624; longitude, 131.584). The isolates formed clearing halos on basal medium agar plates. The strains STUT-A11 and STUT-A16 were selected for genomic analysis and grown aerobically in basal medium overnight at 20°C. The genomic DNA was purified from harvested cells using the NucleoBond high-molecular-weight (HMW) DNA kit (Macherey-Nagel) and subjected to long- and short-read sequencing. For long-read sequencing, genomic DNA (1 μg) was treated with short-read eliminator kit XS (Circulomics) to remove fragments <10 kbp, and libraries were prepared using ligation sequencing kit (Oxford Nanopore Technologies [ONT]). Sequencing was performed on a GridION X5 system using FLO-MIN106 R10.4 and R9.41 flow cells for STUT-A11 and STUT-A16, respectively (ONT). Base-calling was conducted using Guppy v.5.1.13 and v.3.0.3 (ONT), and raw data were filtered (-q 10 -l 1000 --headcrop 50) using NanoFilt v.1.32.1 and v.1.24.0 for STUT-A11 and STUT-A16, respectively (2). For short-read sequencing, libraries were constructed using Nextera DNA Flex library prep kit (Illumina). Paired-end sequencing (2 × 256 bp for STUT-A11 and 2 × 156 bp for STUT-A16) was performed using MiSeq reagent kit v.2 (300 Cycles) on a MiSeq system (Illumina). Raw data were filtered (-q 30 -n 20 -t 1 -T 1 -l 10) using fastp v.0.20.1 and v.0.20.0 for STUT-A11 and STUT-A16, respectively (3). The trimmed long- and short-read data were assembled using Unicycler v.0.4.9b and v.0.4.8 for STUT-A11 and STUT-A16, respectively (4). The assembled sequences were polished using pilon v.1.24 (5); the circularity and absence of structural assembly errors were confirmed using Unicycler and SV-Quest v.1.0 (6), respectively. Genomic annotations were performed using DFAST v.1.2.18 (7, 8) and eggNOG-mapper v.2 (9). Phylogenetic analysis was performed using GTDB-Tk v2.1.0 (10), and average nucleotide identity (ANI) values were calculated using PyANI (11). Default parameters were used for all software unless otherwise specified.

The STUT-A11 genome comprised two circular chromosomes (Table 1). Phylogenetic analysis revealed that the closest relative was Vibrio natriegens NBRC 15636^T^ (NCBI assembly accession no. GCA_001456255) with the pairwise ANI of 90.8%, implying that STUT-A11 represents a newly identified species within the genus *Vibrio*. The genomic sequence of STUT-A16 comprised two circular chromosomes and plasmids (Table 1), with the former displaying ~99% identity to Vibrio diabolicus CNCM I-1629^T^ (assembly accession no. GCA_001048675). However, the strain exhibits a high degree of genomic rearrangement, implying it is a hitherto unknown strain of *Vibrio* species. Genomic annotations based on eggNOG-mapper v2 indicated that STUT-A11 and STUT-A16 chromosomes encode four different β-agarases, α-neoagarooligosaccharide hydrolase, and agarolytic β-galactosidase. The amino acid sequences exhibited >84% identity to similar enzymes in *Vibrio* sp. strain EJY3 (12), and the enzymes may participate in agarose catabolism as in *Vibrio* strain EJY3 (12).

**TABLE 1:** Genomic characteristics of the sequenced isolates

Characteristic	Data for:	
	STUT-A11	STUT-A16
BioSample accession no.	SAMD00529538	SAMD00529539
Short-read sequencing results		
No. of reads (read 1/read 2)	1,585,211/1,585,211	1,166,223/1,166,223
Sum of length (read 1/read 2 [bp])	368,669,617/369,490,141	182,509,425/182,517,996
SRA accession no.	DRR402769	DRR402771
Long-read sequencing results		
No. of reads (bp)	157,633	562,555
Sum of length	1,540,899,968	2,087,099,984
SRA accession no.	DRR402770	DRR402772
Assembly results		
Length (bp)		
Chromosome 1	3,369,721	3,531,696
Chromosome 2	1,884,228	1,881,063
Plasmid 1		6,267
Plasmid 2		3,998
GC content (%)		
Chromosome 1	45.3	44.8
Chromosome 2	44.7	44.6
Plasmid 1		43.4
Plasmid 2		41.8
Avg read depth (×)		
Chromosome 1	158	53
Chromosome 2	108	54
Plasmid 1		1,225
Plasmid 2		4,986
No. of protein-coding sequences^*a*^		
Chromosome 1	2,967	3,128
Chromosome 2	1,651	1,662
Plasmid 1		5
Plasmid 2		3
GenBank accession nos.		
Chromosome 1	AP026763	AP026765
Chromosome 2	AP026764	AP026766
Plasmid 1		AP026767
Plasmid 2		AP026768

a Annotated using DFAST v.1.2.18.

### Data availability.

STUT-A11 and STUT-A16 are associated with BioProject accession no. PRJDB14282, and their accession numbers are provided in Table 1.
